# Suitability of the In Vitro Cytokinesis-Block Micronucleus Test for Genotoxicity Assessment of TiO_2_ Nanoparticles on SH-SY5Y Cells

**DOI:** 10.3390/ijms22168558

**Published:** 2021-08-09

**Authors:** Natalia Fernández-Bertólez, Fátima Brandão, Carla Costa, Eduardo Pásaro, João Paulo Teixeira, Blanca Laffon, Vanessa Valdiglesias

**Affiliations:** 1 Universidade da Coruña, Grupo DICOMOSA, Centro de Investigaciones Científicas Avanzadas (CICA), Departamento de Psicología, Facultad de Ciencias de la Educación, Campus Elviña s/n, 15071 A Coruña, Spain; natalia.fernandezb@udc.es (N.F.-B.); eduardo.pasaro@udc.es (E.P.); 2 Instituto de Investigación Biomédica de A Coruña (INIBIC), AE CICA-INIBIC. Oza, 15071 A Coruña, Spain; vvaldiglesias@udc.es; 3 EPIUnit—Instituto de Saúde Pública, Universidade do Porto, Rua das Taipas, N° 135, 4050-600 Porto, Portugal; fatimabrandao.988@gmail.com (F.B.); carla.trindade@insa.min-saude.pt (C.C.); jpft12@gmail.com (J.P.T.); 4 Environmental Health Department, Portuguese National Institute of Health, Rua Alexandre Herculano 321, 4000-055 Porto, Portugal; 5 Institute of Biomedical Sciences Abel Salazar (ICBAS), University of Porto, Rua de Jorge Viterbo Ferreira 228, 4050-313 Porto, Portugal; 6 Laboratory for Integrative and Translational Research in Population Health (ITR), Rua das Taipas 135, 4050-600 Porto, Portugal; 7 Universidade da Coruña, Grupo DICOMOSA, Centro de Investigaciones Científicas Avanzadas (CICA), Departamento de Biología, Facultad de Ciencias, Campus A Zapateira s/n, 15071, 15071 A Coruña, Spain

**Keywords:** cytochalasin-B, cytokinesis-block micronucleus test, flow cytometry micronucleus test, nanomaterials, TiO_2_ nanoparticles, SH-SY5Y cells, uptake

## Abstract

Standard toxicity tests might not be fully adequate for evaluating nanomaterials since their unique features are also responsible for unexpected interactions. The in vitro cytokinesis-block micronucleus (CBMN) test is recommended for genotoxicity testing, but cytochalasin-B (Cyt-B) may interfere with nanoparticles (NP), leading to inaccurate results. Our objective was to determine whether Cyt-B could interfere with MN induction by TiO_2_ NP in human SH-SY5Y cells, as assessed by CBMN test. Cells were treated for 6 or 24 h, according to three treatment options: co-treatment with Cyt-B, post-treatment, and delayed co-treatment. Influence of Cyt-B on TiO_2_ NP cellular uptake and MN induction as evaluated by flow cytometry (FCMN) were also assessed. TiO_2_ NP were significantly internalized by cells, both in the absence and presence of Cyt-B, indicating that this chemical does not interfere with NP uptake. Dose-dependent increases in MN rates were observed in CBMN test after co-treatment. However, FCMN assay only showed a positive response when Cyt-B was added simultaneously with TiO_2_ NP, suggesting that Cyt-B might alter CBMN assay results. No differences were observed in the comparisons between the treatment options assessed, suggesting they are not adequate alternatives to avoid Cyt-B interference in the specific conditions tested.

## 1. Introduction

The rapid development of nanotechnology has brought huge benefits to our daily lives, but it can also entail potential threats to human health and environment [1,2]. The increasing rate of nanomaterials production and commercialization outpaces the development of adequate tests for evaluating their toxicity, accepted by the scientific community and applicable by regulators. Although the potential risks that nanomaterials exposure may involve have been widely investigated using cell models and a range of in vitro approaches, to date few standardized assays have been validated to accurately assess nanomaterials toxic effects [3]. So far, it is generally accepted that common cell-based toxicity tests might not be fully adequate for evaluating nanomaterials and may not provide reliable data, since the unique features of these materials are also responsible for unexpected interactions with assay components or detection systems [4,5,6,7]. Considering that hazard assessment is a key endpoint to address in management and regulation of manufactured nanomaterials [8,9], appropriate adaptations for nanomaterials in the currently validated protocols to assess potential harmful properties of chemicals are urgently needed to obtain both reliable toxicological profiles and specific standardized methods [10,11,12].

The in vitro mammalian cell micronucleus (MN) test is one of the assays recommended by the Organisation for Economic Cooperation and Development (OECD) for genotoxicity testing of chemicals (Test Guideline (TG) 487) [13], and is considered to be a well-established, reliable, accurate and reproducible endpoint in genotoxicity evaluation [14,15,16]. In fact, this test method has been endorsed as reliable and relevant by the European Centre for the Validation of Alternative Methods (ECVAM) Scientific Advisory Committee (ESAC) [17], and is recommended by the International Council for Harmonization (ICH) and the European Food Safety Authority (EFSA) for genotoxicity testing of pharmaceuticals intended for human use and of food and feed, respectively [18,19]. The OECD TG 487 describes the cytokinesis-block micronucleus (CBMN) assay in different cell models, including primary peripheral blood lymphocytes and a number of rodent and human cell lines. MN are formed from chromosome fragments or whole chromosomes that lag behind during cell division. Excluded from the nuclei of daughter cells, these structures form single or multiple MN in the cytoplasm, which are detected by visual (or automated) microscopic examination after DNA staining [20]. Therefore, the MN assay reveals the ability of substances to induce structural chromosome damage (clastogenic effects) or numerical chromosome alterations (aneugenic effects) [21]. The actin polymerization inhibitor cytochalasin B (Cyt-B) is required in CBMN assay to block cytokinesis and make it possible to differentiate cells that underwent one nuclear division by its binucleate appearance. Nevertheless, Cyt-B may interfere with MN evaluation in nanotoxicology studies mainly by affecting nanomaterials uptake, since many nanomaterials enter the cells by endocytosis-related processes, which involve actin polymerization. This may potentially lead to inaccurate results when this technique is employed for genotoxicity evaluation of nanomaterials [10,14].

Accordingly, among many other NM regulatory and safety assessment issues (i.e., comprehensive physicochemical characterization, cellular uptake assessment, and nanomaterials dispersion protocols), standard genotoxicity tests were discussed in the OECD Working Party on Manufactured Nanomaterials (WPMN) workshop, summarized in report No. 43 [22]. The need to include specific adaptations relevant to testing the genotoxicity of nanomaterials within the existing OECD TG was addressed in the workshop. Recommendations suggested for TG 487 included using p53 competent cell lines, avoid reduced serum concentrations, and applying Cyt-B separately from the nanomaterials treatment using a post-treatment or delayed co-treatment protocol, in order to ensure a period of exposure of the cell culture system to the nanomaterials in the absence of Cyt-B. Moreover, several of these recommendations were also supported by the European Chemicals Agency (ECHA) in the Guidance on information requirements and chemical safety assessment [23]. Besides, measurement of cellular uptake by appropriate methods has been also pointed out as the first key factor to consider for mammalian cell genotoxicity testing, since in some circumstances the absence of genotoxic effects could correspond to the absence of internalization by the cells, and not to the nanomaterials intrinsic safety [24,25].

**Figure 1:**
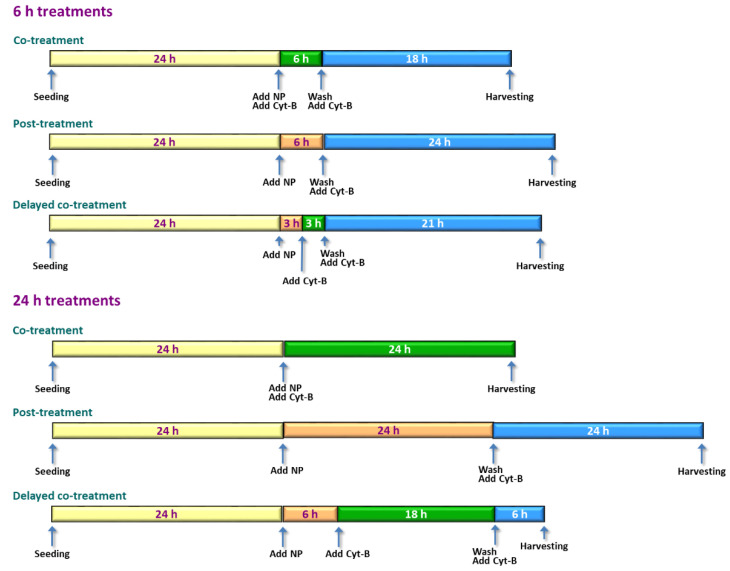
Scheme showing the chronology of CBMN test experiments. Exposure of the cells to TiO_2_ NP was conducted for 6 and 24 h following three different treatment variants: co-treatment, post-treatment, and delayed co-treatment. Color code: yellow: no treatment; orange: treatment with NP; blue: treatment with Cyt-B; green: treatment with NP + Cyt-B.

**Figure 2:**
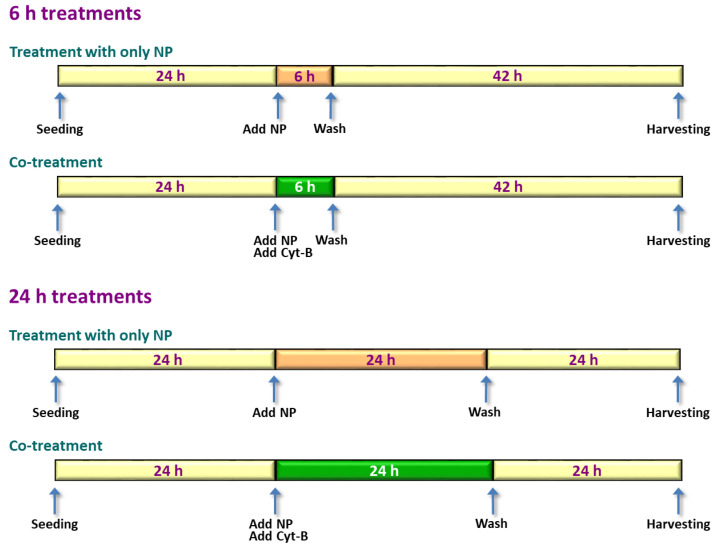
Scheme showing the chronology of FCMN test experiments. Exposure of the cells to TiO_2_ NP was conducted for 6 and 24 h both in the presence and in the absence of Cyt-B. Color code: yellow: no treatment; orange: treatment with NP; green: treatment with NP + Cyt-B.

The main objective of this study was to determine whether the presence of Cyt-B could interfere with titanium dioxide (TiO_2_) nanoparticles (NP) in the assessment of MN induction in human SH-SY5Y neuroblastoma cells by means of the CBMN test. To that aim, CBMN test was conducted in cells treated for 6 or 24 h with TiO_2_ NP, according to three different exposure options: co-treatment (NP and Cyt-B added simultaneously), post-treatment (Cyt-B was added after finishing treatment with NP), and delayed co-treatment (initial exposure to NP for a few hours, and then addition of Cyt-B in co-exposure until the end of NP treatments). Additionally, influence of Cyt-B on the cellular uptake of TiO_2_ NP and on MN induction as evaluated by flow cytometry (FCMN) were also assessed. Figure 1 and Figure 2 show the experimental design of CBMN and FCMN assays experiments, respectively.

## 2. Results

In this study, SH-SY5Y cells were exposed to TiO_2_ NP and Cyt-B under different exposure conditions, and cellular uptake and MN induction (both by means of CBMN test and FCMN test) were assessed. The TiO_2_ NP used were 80% anatase 20% rutile, and their physical–chemical characterization has been previously reported [26]. Their particle size was 25 nm, their specific surface area 35–45 m^2^/g, and their mean hydrodynamic size and zeta potential were 160.5 nm and −27.8 mV in water, and 228.3 nm and −10.7 mV in SH-SY5Y cell culture medium, showing a good dispersion in both media. Regarding cytotoxicity of these particular TiO_2_ NP, no decrease in SH-SY5Y cell viability, as evaluated by 3-(4,5-dimethylthiazol-2-yl)-2,5-diphenyl tetrazolium bromide (MTT) and neutral red uptake assays, was found after treatment with TiO_2_ NP at any concentration (range 20–150 µg/mL) or exposure time (3, 6, and 24 h) [26].

### 2.1. Cellular Uptake

**Figure 3:**
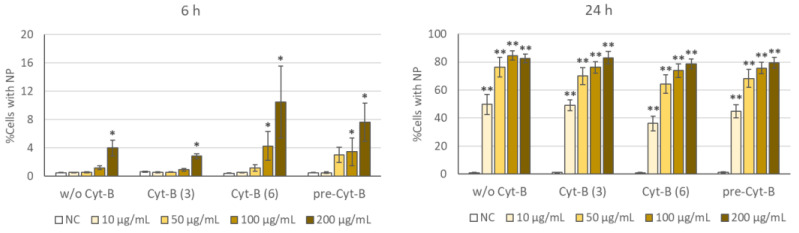
Cellular uptake of the TiO_2_ NP at 6 h (**left**) or 24 h (**right**) treatments. Treatments were conducted in the absence of Cyt-B (w/o Cyt-B), in the presence of 3 or 6 µg/mL of this substance (Cyt-B (3) and Cyt-B (6), respectively), or in the presence of 6 µg/mL Cyt-B added 1 h before the initiation of TiO_2_ NP treatment (pre-Cyt-B). Error bars represent mean standard error. * *p* < 0.05; ** *p* < 0.01, significant difference with regard to the corresponding negative control (NC). No differences were observed in the comparisons between the different treatment options within the same TiO_2_ NP dose and treatment time.

Figure 3 presents the results obtained for the cellular uptake of TiO_2_ NP, as evaluated by flow cytometry. Two exposure times (6 and 24 h) and four different Cyt-B conditions were analyzed: absence of Cyt-B, presence of 3 or 6 µg/mL Cyt-B (simultaneous to the NP treatments), and presence of 6 µg/mL Cyt-B 1 h before NP treatments initiation (maintained up to the end of treatments). Figure S1 shows example dot plots of control cells and cells treated with TiO_2_ NP in the absence and in the presence of Cyt-B. Significant uptake increases were observed at the highest concentrations tested at 6 h, and at all doses at 24 h. Percentages of cells with NP were much more pronounced at the longest exposure time, always higher than 60% from 50 µg/mL on. Still, no significant difference was detected when the different treatment options were compared within the same NP dose and exposure time.

### 2.2. Cytokinesis-Block Micronucleus Test

**Figure 4:**
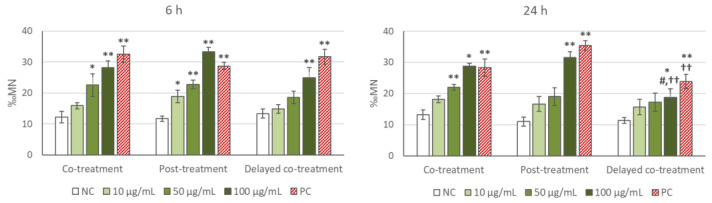
Results of the CBMN test in SH-SY5Y cells treated with TiO_2_ NP for 6 h (**left**) or 24 h (**right**) following three different treatment options: co-treatment, post-treatment, and delayed co-treatment (see Figure 1 for details). Error bars represent mean standard error. * *p* < 0.05; ** *p* < 0.01, significant difference with regard to the corresponding negative control (NC); ^#^ *p* < 0.05, significant difference with regard to 100 µg/mL co-treatment; ^††^ *p* < 0.01, significant difference with regard to the corresponding post-treatment dose. PC: positive control (MMC: 10 µM for 6 h and 1.5 µM for 24 h treatments).

CBMN test results are gathered in Figure 4, for 6 and 24 h treatments. At 6 h, progressive increases in the MN frequencies were obtained in the three treatment options, significant at the highest concentrations (only 100 µg/mL in the case of delayed co-treatment). Dose–response relationships were significant in all cases (co-treatment: r = 0.753, *p* < 0.01; post-treatment: r = 0.894, *p* < 0.01; delayed co-treatment: r = 0.655, *p* < 0.01). No differences were obtained in the comparisons between the treatment options assessed. At 24 h, rising tendencies in the MN rates, significant at the highest doses, also produced significant concentration–effect associations in co-treatment (r = 0.893, *p* < 0.01), and post-treatment (r = 0.771, *p* < 0.01). MN frequencies observed in delayed co-treatment showed also an increasing trend, but with notably lower intensity. In fact, this treatment option showed a significantly lower MN level than the other two options at the highest NP concentration, and than post-treatment in the positive control.

### 2.3. Micronuclei Evaluation by Flow Cytometry

**Figure 5:**
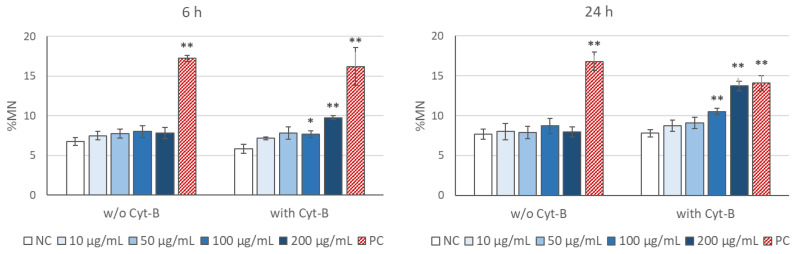
Results of the flow cytometry MN test in SH-SY5Y cells treated with TiO_2_ NP for 6 h (**left**) or 24 h (**right**), in the absence or in the presence of cytochalasin-B (Cyt-B). Error bars represent mean standard error. * *p* < 0.05; ** *p* < 0.01, significant difference with regard to the corresponding negative control (NC). PC: positive control (MMC: 10 µM for 6 h and 1.5 µM for 24 h treatments).

Flow cytometry measurements of MN induction by TiO_2_ NP are depicted in Figure 5. TiO_2_ NP treatments were conducted both in the absence and in the presence of 6 µg/mL Cyt-B, in order to determine whether this chemical compound could interfere with MN production or assessment. In the absence of Cyt-B no significant induction of MN was observed either at 6 h or at 24 h. Nevertheless, significant concentration–effect correlations (r = 0.675, *p* < 0.01 at 6 h; and r = 0.782, *p* < 0.01 at 24 h), with significant increases in MN frequency at the highest doses evaluated was evident in the presence of Cyt-B.

## 3. Discussion

NM that are taken up by mammalian cells can get into direct contact with the genetic material, leading to direct physical or chemical damage, or they can act indirectly (e.g., via inducing oxidative stress) [27]. Genotoxicity in somatic cells can induce carcinogenesis, and accumulation of DNA damage is also associated with various chronic diseases [6]. Consequently, assessing the potential genotoxic properties of substances, including nanomaterials, is a key element in regulatory safety assessment. Several factors affecting evaluation of nanomaterials harmful effects have been previously described for in vitro genotoxicity and cytotoxicity assays, including nanomaterials type, coating and physical-chemical properties, presence of serum proteins in the culture medium, as well as type and origin of cells chosen for testing [27,28,29,30]. In this context, there is a potential need to determine whether methodological adaptations may be required in CBMN assay for a more accurate and reliable measurement of nanomaterials ability to induce chromosome alterations [10,11,31,32,33]. Thus, the main objective of this work was to assess whether results of CBMN test may be influenced by the use of Cyt-B when testing genotoxic effects of TiO_2_ NP on SH-SY5Y cells, and, in such case, to address possible treatment alternatives, in particular those recommended by the OECD WPMN to prevent the interference between Cyt-B and nanomaterials.

TiO_2_ is the most widely used nanomaterial worldwide, employed in a variety of applications ranging from medical devices to products used in everyday life [31,34]. The present work was carried out using TiO_2_ NP as nanomaterial model since their genotoxicity has been extensively studied and they are known to induce MN in a variety of cell lines [12,35,36,37,38]. Moreover, following OECD TG 487 and OECD report No. 43, MN test was conducted in cells of human origin, specifically in SH-SY5Y cells. This cell line has been broadly used as cellular model in neurobiological, neurochemical and neurotoxicological evaluations [39,40,41], and they are p53 proficient [42]. Furthermore, they were previously demonstrated to effectively internalize TiO_2_ NP [26,43].

Prior to genotoxicity testing, cellular uptake of TiO_2_ NP in the presence or absence of Cyt-B at different conditions was evaluated by flow cytometry analysis. The CBMN assay relies on the use of Cyt-B to prevent cytokinesis by inhibiting actin assembly, generating binucleated cells in which the MN frequency is scored. When nanomaterials are evaluated, this methodological procedure could cause a potential problem as Cyt-B also inhibits endocytosis processes—in which actin plays an essential role [44]—used by most cells to internalize nanomaterials [10,45]. Therefore, evaluating whether the presence of Cyt-B interferes with the cellular uptake of study NP is essential and very useful to interpret correctly the CBMN results obtained for these NP [6,46,47,48,49]. In the present study, cellular uptake results confirmed an efficient dose- and time-dependent internalization of the TiO_2_ NP by SH-SY5Y cells, regardless the Cyt-B concentration or its presence in the medium prior to the TiO_2_ NP treatments. Agreeing with our positive results, a number of previous studies have proved that TiO_2_ NP, either single particles or particle aggregates, are rapidly internalized by different types of cells, both of rodent and human origin, and this uptake was also demonstrated to be dose- and time-dependent [40,50,51,52].

It was previously reported that NP internalization in cells occurs mainly through phagocytosis or macropinocytosis [47,53]. These endocytic processes are fundamentally determined by size, shape, specific surface area and crystalline structure of the particles, as well as by NP concentration and state of aggregation/agglomeration, incubation time, and intrinsic endocytic properties of the cell type exposed [27]. The absence of influence of Cyt-B on cellular uptake of TiO_2_ NP observed in our study, at concentrations effective to inhibit cytokinesis, suggest that NP are internalized by an actin-independent process. Indeed, although TiO_2_ NP were reported to be internalized by endocytic pathway in some cell types [54,55], other studies demonstrated that the uptake of these NP vary depending not only on the cell type but also on TiO_2_ NP agglomeration, since small NP likely cross the cellular membrane directly, whereas agglomerated NP would need to be taken up by endocytosis [50,56,57]. Accordingly, the mean hydrodynamic size of TiO_2_ NP used in this study (228.3 nm), their zeta potential (−10.7 mV), and their size distribution in SH-SY5Y culture medium [26], indicating relatively small size and little agglomeration, support a non-actin mediated uptake for these NP in the conditions tested.

In the present study, treatment of the cells with the TiO_2_ NP was the same in both MN evaluation techniques (FCMN and CBMN): for 6 and 24 h, but in CBMN three different exposure variants were tested, according to the moment Cyt-B was added to the culture (co-treatment, post-treatment, and delayed co-treatment). In turn, in FCMN test, cells were treated either in the absence or in the presence (co-treatment) of Cyt-B, in order to test whether Cyt-B may influence in some way MN induction by the NP. Results obtained from standard CBMN assay, i.e., with co-treatment of NP and Cyt-B, showed that exposure to TiO_2_ NP induced dose-dependent MN increase in SH-SY5Y cells, statistically significant at the highest concentrations, after both 6 and 24 h treatments. However, such MN induction was not detected by flow cytometry (methodology that do not require Cyt-B), and only a dose-dependent positive response was obtained when this chemical was intentionally added simultaneously with TiO_2_ NP to force the co-treatment. The flow cytometry MN evaluation involves scoring MN frequency in mononuclear cells (Cyt-B is therefore not necessary), and has been widely used and validated for genotoxicity testing of a variety of chemicals [58,59,60,61].

Numerous studies obtained concordant results between in vitro MN evaluation by CBMN and FCMN assays, both treating with chemicals [62,63] and with NP, particularly with TiO_2_ NP [51,64]. Considering this, the dissimilar results found in our study between CBMN assay and FCMN test (in the absence of Cyt-B treatment) would indicate that Cyt-B may indeed be altering the results of CBMN assay by increasing MN frequency in SH-SY5Y cells, leading to false positive results in the conditions tested. On the basis of our data it seems that, although Cyt-B does not interfere with TiO_2_ NP cellular uptake, it could affect other processes such as the intracellular transport of particles, vesicles or organelles, inducing changes in the subcellular NP location, as previously observed by other authors [54,55,57,65]. Previous CBMN studies on MN induction by TiO_2_ NP in co-treatment with Cyt-B (4–9 µg/mL) showed both positive and negative results [66,67,68], depending on factors such as treatment time or NP size, but also NP crystalline structure (anatase, rutile, and mixed form), as only anatase phase particles increased the MN frequency [67,68]. However, these studies did not explore exposure options different from co-treatment, or additional evaluation by FCMN test.

Furthermore, it seems surprising that MN frequencies induced by TiO_2_ NP, as evaluated by CBMN test, were similar at 6 and 24 h treatments, when cellular uptake was much lower at 6 h than at 24 h. Considering that MN induction by TiO_2_ NP was only observed by FCMN in the presence of Cyt-B, and that Cyt-B concentration was the same at both treatment times (6 µg/mL), this may indicate that the presence of Cyt-B is not only affecting MN results, but also determining MN induction to a higher extent than NP internalization by cells.

Apart from the standard co-treatment in CBMN test, in which interference of Cyt-B with TiO_2_ NP treatment was evidenced, the two possible treatment alternatives recommended by OECD WPMN to prevent this interference—i.e., post-treatment and delayed co-treatment [22]—were also carried out in order to check their suitability for MN evaluation and, if applicable, to provide evidence of the best option. Studies in the literature comparing the standard co-treatment experimental condition in the CBMN test with the two other treatment options recommended for nanomaterials testing are scarce [45,69]. Our results showed no differences between post-treatment and co-treatment, and only a slight decrease in MN frequency in delayed co-treatment, limited to the highest concentration and the longest treatment tested, with regard to the other two treatment options. Moreover, the fact that the decrease in MN rate after 24 h of delayed co-treatment was also observed in the positive control (MMC), rules out the possibility that this can be due to a lower interference between NP and Cyt-B. Therefore, results observed in these experiments indicate that the two treatment options recommended for testing nanomaterials by the CBMN assay are not good options in this particular case, since they resulted similar than the standard co-treatment, and the data obtained substantially differed from those coming from FCMN test in the absence of Cyt-B. Following the recommendations provided by OECD WPMN [22], a number of researchers evaluated MN induction by TiO_2_ NP exposure applying either post-treatment [12,70,71,72] or delayed co-treatment [51,55,73,74] in a number of different cell types. Similar to our results, these studies frequently found increases in MN frequency after TiO_2_ NP exposure. Nevertheless, it is not possible to determine whether the presence of Cyt-B is influencing NP uptake and/or MN rates in these studies, since no comparisons were carried out with other alternative protocols or methodologies.

## 4. Materials and Methods

### 4.1. Chemicals

Titanium dioxide (TiO_2_ NP) (CAS No. 13463-67-7, size 25 nm) was purchased from Degussa-Evonik (Bitterfeld, Germany), mitomycin C (MMC) (CAS no. 50-07-7), cytochalasin B (Cyt-B) (CAS no. 14930-96-2), dimethyl sulfoxide (DMSO) (CAS no. 67-68-5), 4,6-diamino-2-fenilindol (DAPI) (CAS no. 28718-90-3), propidium iodide (PI) (CAS no. 25535-16-4), and ribonuclease (RNase) A from bovine pancreas (CAS no. 9001-99-4) were purchased from Sigma-Aldrich Co. (Madrid, Spain). MMC was dissolved in sterile distilled water, and Cyt-B was dissolved in DMSO.

### 4.2. Preparation of Nanoparticle Suspension

TiO_2_ NP suspensions were prepared following the same protocol employed in previously published studies [26,43]. Briefly, TiO_2_ NP were suspended in either deionized water or cell culture medium (see composition below) at a final concentration of 200 µg/mL and ultrasonicated (Branson Sonifier, Carouge Switzerland) at 30 W for 5 min (1.5 min on and 1 min off twice, plus 2 min on). To prevent heating of the NP suspension, it was maintained in ice during the sonication procedure.

### 4.3. Cell Culture and Treatments

Human neuroblastoma SH-SY5Y cell line was obtained from the European Collection of Authenticated Cell Cultures (ECACC94030304). Cell culture medium consisted of a nutrient mixture EMEM/F12 (1:1) medium with 1% non-essential amino acids, 1% antibiotic and antimycotic solution, and supplemented with 10% heat-inactivated fetal bovine serum (FBS) (all from Gibco, Thermo Fisher Scientific Inc., Uppsala, Sweden). Cells were grown in a humidified atmosphere with 5% CO_2_ at 37 °C. They were seeded in 96-well plates (5–6 × 10^4^ cells/well) and allowed to adhere for 24 h at 37 °C prior to the experiments. For each uptake and FCMN experiment, cells were incubated with four different concentrations of TiO_2_ NP (10, 50, 100, and 200 μg/mL) at 37 °C, at a final volume of 100 μL per well in 96-well plates. For CBMN experiments, cells were incubated at 10, 50, or 100 μg/mL TiO_2_ NP (37 °C), at a final volume of 1 mL per well in 24-well plates. The highest TiO_2_ NP concentration (200 μg/mL) was discarded for these experiments since the NP density interfered with the microscopic visual scoring of MN. Cell culture media was used as negative control, and MMC (10 µM for 6 h experiments and 1.5 µM for 24 h experiments) was used as positive control for CBMN and FCMN tests experiments.

### 4.4. Cellular Uptake

For analysis of cellular uptake, SH-SY5Y cells (6 × 10^4^ cells/mL) were exposed to TiO_2_ NP for 6 and 24 h. For each treatment time, four TiO_2_ NP treatment conditions were tested as follows: absence of Cyt-B, simultaneous presence of 3 or 6 µg/mL Cyt-B, and pre-incubation with 6 µg/mL Cyt-B for 1 h before adding the TiO_2_ NP [75]. Distilled water or 1% DMSO in EMEM/F12 (1:1) supplemented medium were employed as negative controls for experiments without or with Cyt-B, respectively. When treatments were finished, cells were then centrifuged at 660× *g*, trypsinized (100 µL trypsin 0.05%, Gibco, Thermo Fisher Scientific Inc., Uppsala, Sweden), transfered to a 5 mL tube with 900 µL phosphate buffered saline (PBS), centrifuged again, and the cellular pellet was resuspended in 300 µL of cytometry-grade PBS for analysis. Internalization of the NP by the SH-SY5Y cells was evaluated by flow cytometry following Suzuki et al. [76], using a FACSCalibur flow cytometer (Becton Dickinson, Madrid, Spain). After finishing the treatments, cell populations were analyzed using CellQuest Pro software (Becton Dickinson, Madrid, Spain), on the basis of their size (forward scatter signal, FSC), and complexity (side scatter signal, SSC).

### 4.5. Cytokinesis-Block Micronucleus Test

Cells were seeded at a cell density of 4 × 10^4^ cells/well and incubated at 37 °C for 24 h in a humidified atmosphere with 5% CO_2_. After this period, cells were treated for 6 or 24 h with the dose range of TiO_2_ NP. Negative controls (culture medium) and positive controls (10 or 1.5 µM MMC for 6 or 24 h experiments, respectively) were added for each experimental variant. At this point, CBMN test was conducted following three different conditions of exposure to TiO_2_ NP and Cyt-B (6 µg/mL) for each treatment time (6 and 24 h) (Figure 1): (i) co-treatment: simultaneous exposure to TiO_2_ NP and Cyt-B (in the 6 h treatments NP are washed out after this time, and Cyt-B remained up to 24 h; in the 24 h exposure both compounds remained for the whole treatment duration); (ii) post-treatment: initial exposure to TiO_2_ NP for the corresponding treatment time, washing out of the NP, addition of Cyt-B and incubation for an additional 24 h period; (iii) delayed co-treatment: initial exposure to the TiO_2_ NP for 3 h (in the 6 h treatments) or 6 h (in the 24 h treatments), then addition of Cyt-B and incubation for an additional 24 h period (NP were removed at the end of the corresponding treatment time).

When all cultures were finished (treated with TiO_2_ NP, negative and positive controls), the medium with Cyt-B was removed and cells were washed twice with PBS and harvested with 250 µL of trypsin 0.05% (Gibco, Thermo Fisher Scientific Inc., Uppsala, Sweden) (incubated for 3 min at 37 °C). The trypsin was inactivated with 500 µL of PBS and cells were transferred to a 15 mL tube; the wells were washed with additional 250 µL of PBS which were then added to the respective tube. Cells were centrifuged at 200× *g* for 10 min, the supernatant was removed, and cells were re-suspended in 4 mL of Carnoy fixative (methanol-acetic acid, 3:1). After a new centrifugation at 200× *g* 10 min, pellets were resuspended in a small volume of Carnoy and dropped onto clean slides, that were air dried overnight. Slides were stained with 5 µg/mL 4,6-diamidino-2-phenylindole (DAPI). Scoring was performed with a Leica DM-RXA fluorescence microscope, equipped with a 100 W mercury lamp and a 100× magnification objective. A total of 2000 binucleated cells with well-preserved cytoplasm, half from each duplicate culture, were blindly scored by the same reader to determine the number of MN. Criteria described by Fenech et al. [77] were followed for identifying binucleated cytokinesis-blocked cells and MN.

### 4.6. Micronuclei Evaluation by Flow Cytometry

For the analysis of MN induction by flow cytometry (FCMN), cells were seeded at 4 × 10^4^ cells/well and incubated for 24 h at 37 °C in a humidified environment with 5% CO_2_. TiO_2_ NP treatments were conducted both in the absence and in the presence of 6 µg/mL Cyt-B (see experimental design in Figure 2). When the 6 and 24 h treatments were finished, SH-SY5Y cells were cultured in fresh medium for an additional period until completing 48 h, time determined on the basis of cell cycle duration. When this period was finished, the medium was removed and all cells (treated with TiO_2_ NP, negative and positive controls) were washed twice with PBS and harvested with 100 µL of trypsin 0.05% (Gibco, Thermo Fisher Scientific Inc., Uppsala, Sweden) for 3 min at 37 °C. Then they were transferred to a 5 mL tube containing 900 µL of PBS, split into the two replicates, and subsequently a suspension of nuclei and MN was prepared according to the procedure described by Nüsse et al. [78], with some modifications [63]. Briefly, cells were centrifuged at 660× *g* for 5 min, and the cell pellet was resuspended in solution I (584 mg/L sodium chloride, 1 g/L sodium citrate, 50 μg/mL PI, 50 μg/mL RNase A, and 0.3 mL/L Nonidet *p*-40) to yield a final concentration of 106 cells/mL. Samples were maintained in the dark at room temperature for 30 min. Then an equal amount of solution II (15 mg/L citric acid and 85.6 g/L sucrose) was added and cells were incubated for 15 min in the dark at room temperature. Finally, the suspension was filtered through a 50 μm nylon mesh, and flow cytometry measurements were carried out. This two-step procedure destroyed the cell membrane and cytoplasm, thus the suspension contained relatively pure nuclei and MN. This suspension was analyzed with a FACSCalibur flow cytometer (Becton Dickinson, Madrid, Spain). For each sample, a minimum, 50,000 events were evaluated from the PI signal. Data analysis was conducted with Cell Quest Pro software (Becton Dickinson, Madrid, Spain), following the instructions described by Avlasevich et al. [59].

### 4.7. Statistical Analysis

A minimum of three independent experiments and two replicates per experiment were performed for each experimental condition tested. Experimental data were expressed as mean ± mean standard error. Kruskal–Wallis test was applied for comparisons among groups and Mann–Whitney *U*-test to analyze differences in two-by-two comparisons. Associations between two variables were analyzed by Spearman’s correlation. The probability level of 0.05 was used as the criterion of significance. All statistical analyses were conducted using the IBM^®^ SPSS^®^ software package V. 21 (Madrid, Spain).

## 5. Conclusions

Despite previous studies highlighted the possible interference of Cyt-B on NP cellular uptake and, consequently, on MN detection by CBMN test, current data do not support that hypothesis in the case of SH-SY5Y cells exposed to TiO_2_ NP. Cyt-B did not affect the NP uptake at any condition tested. Consequently, the discordant results obtained between CBMN and FCMN assays, likely due to the presence of Cyt-B used to block cytokinesis in CBMN test, cannot be attributed to the lack of NP internalization found in other studies. Post-treatment and delayed co-treatment of Cyt-B proposed by OECD WPMN and tested in this study seem not to be adequate alternatives to avoid this interference under the specific conditions employed. Consequently, further investigations are still necessary to address this interference and define additional protocol alternatives of CBMN assay for accurately assessing genotoxicity of nanomaterials.

## Data Availability

The data presented in this study are available from the corresponding author on request.

## Supplementary Materials

The following are available online at https://www.mdpi.com/article/10.3390/ijms22168558/s1, Figure S1: Flow cytometry dot plots of cellular uptake analysis of SH-SY5Y cells after 6 h (a–c) and 24 h (d–f) treatments. (a,d) Control cells; (b,e) cells treated with 100 µg/mL TiO_2_ NPs in the absence of Cyt-B; (c,f) cells treated with 100 µg/mL TiO_2_ NPs in the presence of 6 µg/mL Cyt-B. R2: Region of cells containing NPs.
