# Supplementary material for: Suitability of the In Vitro Cytokinesis-Block Micronucleus Test for Genotoxicity Assessment of TiO2 Nanoparticles on SH-SY5Y Cells
Source: Int J Mol Sci. 2021 Aug 9;22(16):8558. doi: 10.3390/ijms22168558 (PMC8395234; doi:10.3390/ijms22168558)
Supplement: Supplementary file 1 [file ijms-22-08558-s001.zip › ijms-1250923-supplementary.pdf]

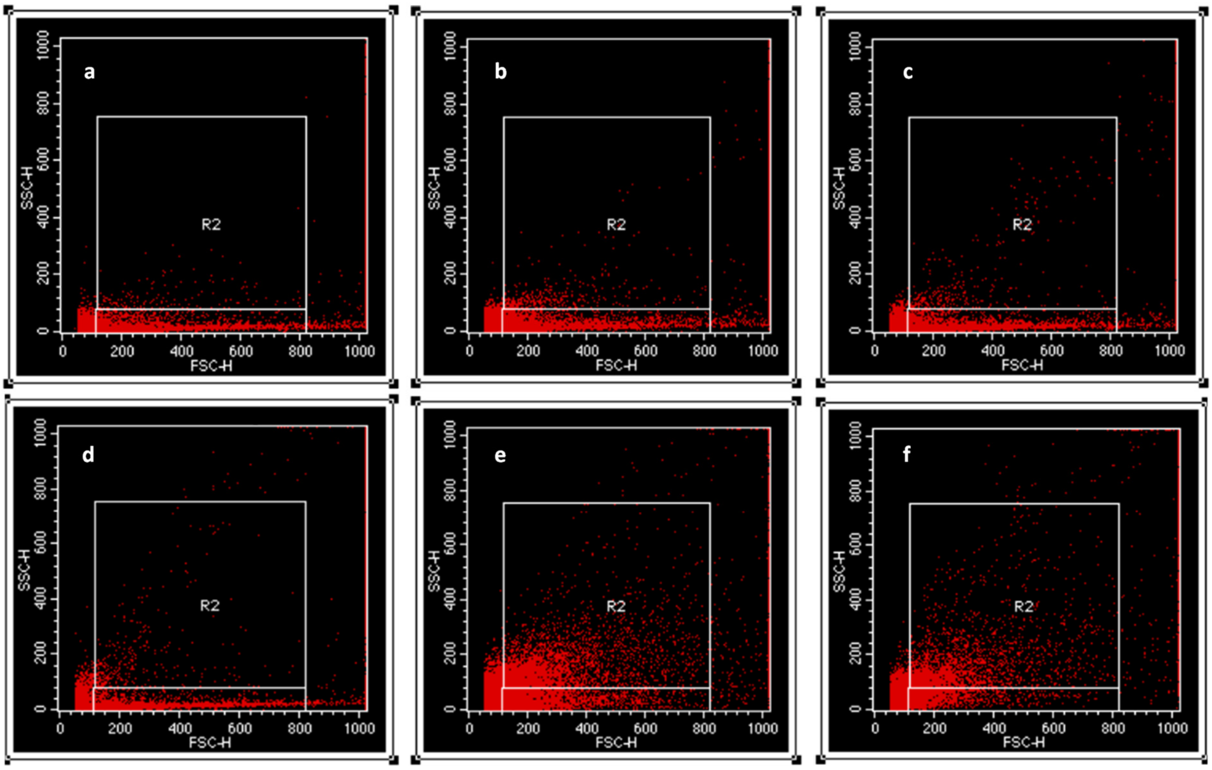

Figure S1. Flow cytometry dot plots of cellular uptake analysis of SH-SY5Y cells after 6 h (a-c) and 24 h (d-f) treatments. (a,d) Control cells; (b,e) cells treated with 100 μg/mL TiO_2 NPs in the absence of Cyt-B; (c,f) cells treated with 100 μg/mL TiO_2 NPs in the presence of 6 μg/mL Cyt-B. R2: Region of cells containing NPs.
